# Accuracy of cone-beam computed tomography for the evaluation of mandible invasion by oral squamous cell carcinoma

**DOI:** 10.1186/s12903-021-01567-3

**Published:** 2021-05-01

**Authors:** Zezheng Wang, Shuang Zhang, Yumei Pu, Yuxin Wang, Zitong Lin, Zhiyong Wang

**Affiliations:** Nanjing Stomatological Hospital, Medical School of Nanjing University, Nanjing, 210008 China

**Keywords:** Accuracy, Cone-beam computed tomography, Mandible invasion, Oral squamous cell carcinoma

## Abstract

**Background:**

For patients with oral squamous cell carcinoma (OSCC), accurate evaluation of mandible invasion and resection with appropriate boundaries are important for preserving the structure and function of the mandible and preventing local recurrence. Although cone-beam computed tomography (CBCT), which has high spatial resolution, is now widely used in the diagnosis of oral and maxillofacial bone lesions, no studies have systematically evaluated the accuracy of CBCT for evaluating the presence of bone invasion, the boundaries of bone invasion and the presence of nerve invasion. Therefore, this study aimed to systemically explore the accuracy of CBCT in the preoperative assessment of mandibular invasion by OSCC.

**Methods:**

Thirty mandibular specimens from OSCC patients were collected in this study. The samples were marked and subjected to CBCT examination. Hematoxylin–eosin staining was used for histopathological assessment sed as the gold standard. The evaluation included the presence of bone invasion, the boundaries of bone invasion and the presence of nerve invasion. The CBCT and histopathological boundaries of bone invasion were delineated and merged to compare and calculate the deviation of CBCT in boundary evaluation.

**Results:**

The accuracy of CBCT in evaluating the presence of mandible invasion was 100%, and the accuracy of CBCT in evaluating the presence of nerve invasion was 69.2%. A mean deviation of 2.97 mm was found for assessment of the boundary of bone invasion using CBCT compared with the histopathological standard. The interexaminer agreement and intraexaminer agreement were perfect for the detection of bone invasion and nerve invasion (κ > 0.8). The intraclass correlation coefficient was 0.923 for the consistency test of boundary delineation on CBCT images.

**Conclusion:**

CBCT is quite reliable in determining the presence or absence of mandible invasion but not as reliable for nerve invasion. The deviation in bone invasion boundary estimation should be considered in osteotomy for OSCC.

## Background

Oral squamous cell carcinoma (OSCC) with mandible invasion is associated with a poor prognosis [1]. Determination of the presence and extent of mandible invasion in a patient with OSCC is important for ensuring complete resection of lesions with clear margins and for planning mandibular reconstruction [2]. However, intraoperative determination of the surgical margin is almost impossible because of the high mineral content of the involved tissue and the time-consuming pathological decalcification procedure. Currently, surgeons rely on preoperative clinical and radiographic examinations to determine the presence or absence of bone invasion and the extent of mandibular resection required [3]. To ensure adequate resection, marginal or segmental mandibulectomy is performed for lesions with radiographic confirmation of mandible invasion [4]. Moreover, to ensure the maximum preservation of tissue and function, the boundary of the resected mandible should be determined precisely. Given the benefits of achieving complete resection with clear margins while achieving maximum preservation of the unaffected tissue, an accurate preoperative assessment of mandibular invasion by OSCC is crucial for optimizing treatment and preventing local recurrence.

Cone-beam computed tomography (CBCT), which has high spatial resolution and a low cost, is currently widely used in the diagnosis of oral and maxillofacial bone lesions. The most commonly used resolution of CBCT for maxillofacial bone lesions is 0.25 mm, which is much smaller than that of conventional 64-slice spiral CT (1 mm). It has been found that CBCT has high accuracy in evaluating the presence or absence of mandible invasion by OSCC. However, no studies have investigated the accuracy of this modality for predicting the extent of invasion.

The present study aimed to explore the accuracy of using CBCT to evaluate mandible invasion by OSCC, with histopathological examination as the gold standard. The presence or absence of bone invasion, the boundary of bone invasion and inferior alveolar nerve invasion were evaluated, and high consistency was found between the CBCT evaluation and the final pathological results. CBCT was found to be useful and accurate in the preoperative evaluation of mandible invasion by OSCCs.

## Methods

This study was approved by the Human Ethics Committee of the Medical School of Nanjing University, Nanjing Stomatological Hospital, China. All subjects signed an informed consent form after receiving a detailed explanation of the study. This study followed the Standards for Reporting Diagnostic Accuracy (STARD) recommendations [5].

### Sample acquisition and pretreatment

**Fig. 1:**
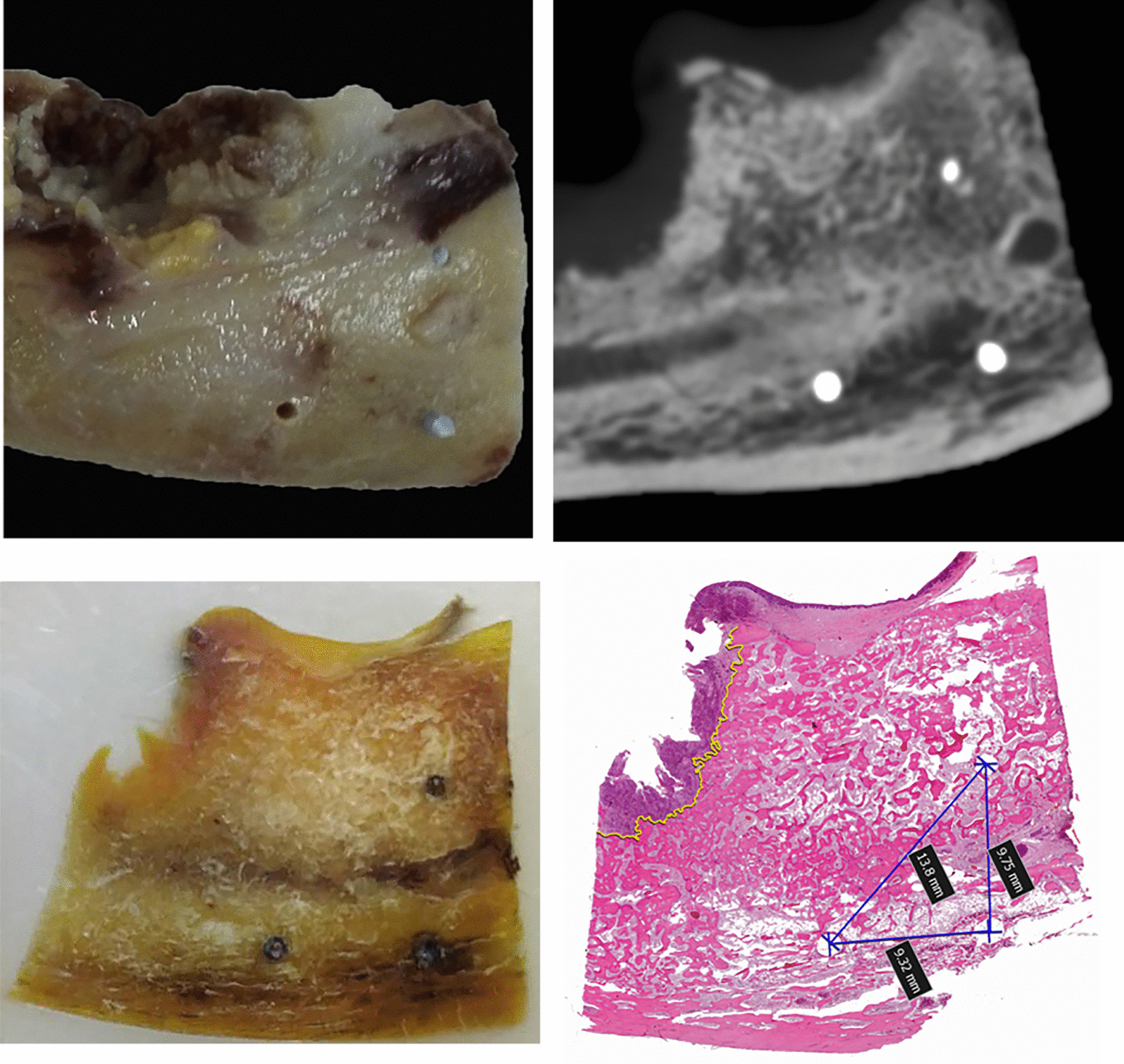
**a** Three gutta-percha points were placed in the samples. **b** Gutta-percha points appeared as highlights in a cone-beam computed tomography image. **c** Simultaneous surface exposure of three gutta-percha points. **d** A section including three gutta-percha points was stained with hematoxylin–eosin; subsequent dissolution of the points yielded three round holes

Thirty mandibular specimens were collected from 30 Chinese patients who were pathologically diagnosed with OSCC at the Nanjing Stomatology Hospital between June 2015 and June 2017. Surgeries were performed according to the protocol in which the involved bone was resected by marginal resection when the tumor was adjacent to or fixed to the mandible and by segmental resection in cases of mandibular invasion. No patient received preoperative radiotherapy or chemotherapy. All the samples were implanted with 3 gutta-percha (GP) points, which served as calibration points in the subsequent evaluation using CBCT images. Three 1-mm holes were drilled into the cancellous bone with a depth of approximately 4–6 mm, which ensured that the bottom of the hole was in the cancellous bone. Then, three 1-mm GP points were inserted into the bottom of each hole (Fig. 1a). Because GP points are firmly inserted into the three holes, the location of the three GP points would remain still during subsequent processing. These three GP points were used as location points, which enabled comparison of CBCT images and pathological slices. The distances between the GP points were also measured to calculate the ratio of tissue shrinkage.

### CBCT image acquisition and assessment

Then, all specimens underwent CBCT scanning with the same CBCT scanner (KaVo 3D eXam, USA) according to the manufacturer’s instructions. The scanning parameters were as follows: voxel size: 0. 25 mm; field of view: 16 × 13 cm; tube voltage: 120 kVp, and tube current: 5.0 mA. eXamVision 1.6 software (KaVo 3D eXam, USA) was used to evaluate and delineate the boundaries of bone invasion. First, CBCT images marked by the three GP points were found (Fig. 1b). Then, negative or positive bone invasion and inferior alveolar nerve invasion were evaluated on these CBCT images. Decreased bone density of medullary bone and/or discontinuous cortical bone was diagnosed as positive for bone invasion. Discontinuity of the inferior alveolar nerve canal was diagnosed as nerve invasion.

Two observers assessed all images twice and determined the diagnostic result independently. If a consensus could not be reached between the 2 examiners, a senior radiologist determined the final result. Before evaluation, calibration, including unified training on diagnostic standards for bone invasion and nerve invasion, was performed. Subsequently, a senior radiologist delineated the boundary of bone invasion for those positive samples. Three months later, the senior radiologist assessed the CBCT images and delineated the boundary of bone invasion again to evaluate the reproducibility of the study. None of the observers had any clinical information about these samples.

### Histopathological assessment

After scanning with CBCT, specimens were immediately fixed in 4% formaldehyde and preserved for at least 48 h. Subsequently, they were decalcified in a mixture of formic acid, acetic acid, and hydrochloric acid. During decalcification, the superficial bone became soft and was removed. This process was repeated until a 3-mm-thick bone slice with the three GP points exposed on the surface was acquired (Fig. 1c). The whole decalcification process took 7–14 days. Finally, the specimens were sliced into 4-μm-thick sections and stained with hematoxylin–eosin. The GP points dissolved during the staining process, and blank circles served as markers of their original positions.

The stained sections were scanned using a digital slice scanning device (NanoZoomer S60, Japan), and the boundary of bone invasion was delineated using NDP. View 2.7 software (Hamamatsu, Japan). The distances between the GP points were measured for specimens embedded in paraffin and specimens stained with hematoxylin–eosin. For histopathological specimens, bone invasion was defined as replacement of bone by an advancing tumor front. The presence of tumor cells in the inferior alveolar nerve fibers indicated positive nerve invasion. A senior pathologist determined the diagnostic result and delineated the boundary of bone invasion with NDP. View 2.7 software (Hamamatsu, Japan).

### Fusion of CBCT and pathological images

The histopathological images were fused with the CBCT images using Adobe Photoshop CC 14.0 software (Adobe, USA) to evaluate the consistency of the boundary of bone invasion on CBCT images and pathological images. The pathological images were magnified to enable overlapping of the three GP points completely due to bone shrinkage that occurred during the bone decalcification process. The difference in the boundary of bone invasion on CBCT images and pathological images was measured with the ruler tool of Adobe Photoshop CC, and the actual difference was calculated according to the scale.

### Statistical analysis

Cohen’s kappa (κ) statistics were used to assess inter- and intraexaminer agreement for the detection of bone invasion and nerve invasion. The intraclass correlation coefficient (ICC) was used for the consistency test of the two boundary delineations on the CBCT image. Using the histopathological results as the gold standard, the test efficiency of CBCT for mandible invasion was calculated. The distances between the GP points measured on CBCT images and histological images were compared by paired t-tests. All statistical analyses were performed using SPSS 23 (IBM SPSS Statistics Base Integrated Armonk, NY, USA). Data were analyzed as originally recorded, without missing data handling.

## Results

Among the 30 specimens, 15 (50%) were obtained from patients who underwent segmental mandibulectomy, and the remaining 15 (50%) were obtained from patients who underwent marginal mandibulectomy. Of the 15 segmental mandibulectomy specimens, 13 (87%) specimens presented with mandible invasion, and 6 specimens presented with nerve invasion (Table 1).

**Table 1:** Patient characteristics and diagnostic accuracy by cone-beam computed tomography

	Gender			Age (years)			TP	TN	FP	FN	Sensitivity (%)	Specificity (%)	Accuracy (%)
	N	M	F	21–40	41–60	61–80							
Bone invasion^a^	30	19 (63.3%)	11 (36.7%)	1	18	11	13	27	0	0	100	100	100
Nerve invasion^b^	13	8 (61.5%)	5 (38.5%)	0	6	7	4	5	2	2	66.7	71.4	69.2

N, the total number of patients; M, males; F, females; TP, true positive; TN, true negative; FP, false positive; FN, false negative

^a^Diagnostic accuracy of cone-beam computed tomography for the detection of bone invasion by oral squamous cell carcinoma

^b^Diagnostic accuracy of cone-beam computed tomography for the detection of inferior alveolar nerve invasion by oral squamous cell carcinoma. The samples were from bone invasion samples

### Tissue shrinkage

**Fig. 2:**
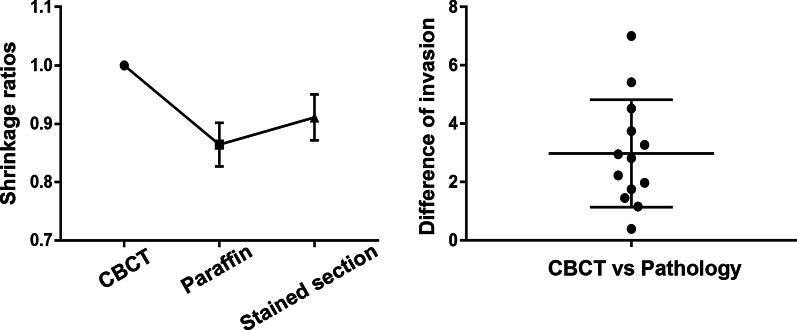
**a** Tissue shrinkage ratios during histological processing. Tissue shrinkage occurred during paraffin embedding and was reversed during subsequent processing. **b** The largest difference in the invasive front between CBCT and histopathological images. This largest difference ranged between 0 and 7 mm in each specimen, with an average difference of 2.97 mm. CBCT, cone-beam computed tomography

The distances between the GP points measured on histological slices were significantly smaller than those measured on the CBCT images, suggesting significant tissue shrinkage due to histological processing. Moreover, the distances measured after hematoxylin–eosin staining were larger than those measured after paraffin embedding. The tissue shrinkage ratios determined from histological and CBCT measurements are shown in Fig. 2a. The overall tissue shrinkage ratio was 91.1% (95% confidence interval: 90.2–92.0%).

### Accuracy of CBCT in the diagnosis of mandible invasion

**Fig. 3:**
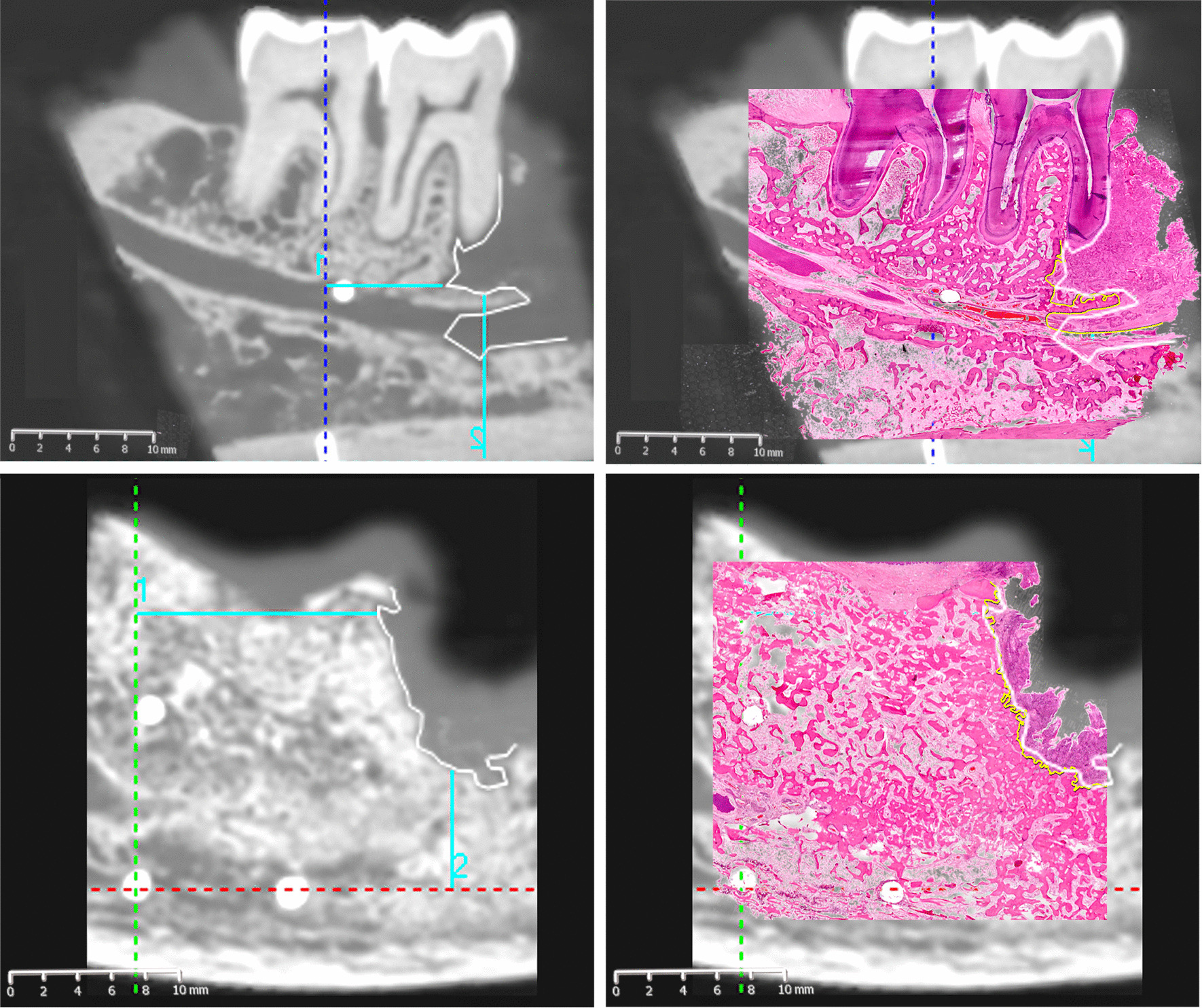
Merged cone-beam computed tomography and histopathological images depicting the tumor borders determined by each modality. **a** Mandibular invasion with involvement of the inferior alveolar nerve canal. **b** Mandibular invasion distant from the inferior alveolar nerve. The white and yellow lines indicate the borders delineated by cone-beam computed tomography and by histopathological examination, respectively

There was perfect interexaminer agreement for the detection of bone invasion (κ = 1) and the detection of nerve invasion (κ = 1) and high intraexaminer agreement for the detection of bone invasion (κ = 1) and the detection of nerve invasion (κ = 0.843). Using the pathological boundary as a reference, the boundary difference values measured by the two delineations on CBCT images were highly correlated (ICC = 0.923). For bone invasion, the diagnosis was accurate for all the negative and positive samples using CBCT; the sensitivity, specificity and accuracy were all 100%. Of the 6 positive nerve invasion samples, 4 were true positive and 2 were false negative, and the sensitivity, specificity and accuracy were 66.7%, 71.4% and 69.2%, respectively (Table 1). A visual observation of the merged images revealed differences in the extent of invasion determined via histopathological examination and CBCT. Figure 2b depicts the most significant difference for each specimen. CBCT tended to underestimate the extent of invasion relative to that determined via histopathological examination, with an average difference of 2.97 mm (95% confidence interval: 1.86–4.09 mm). Figure 3 shows the matched tumor borders delineated by the two methods.

### Pattern of mandible invasion

**Fig. 4:**
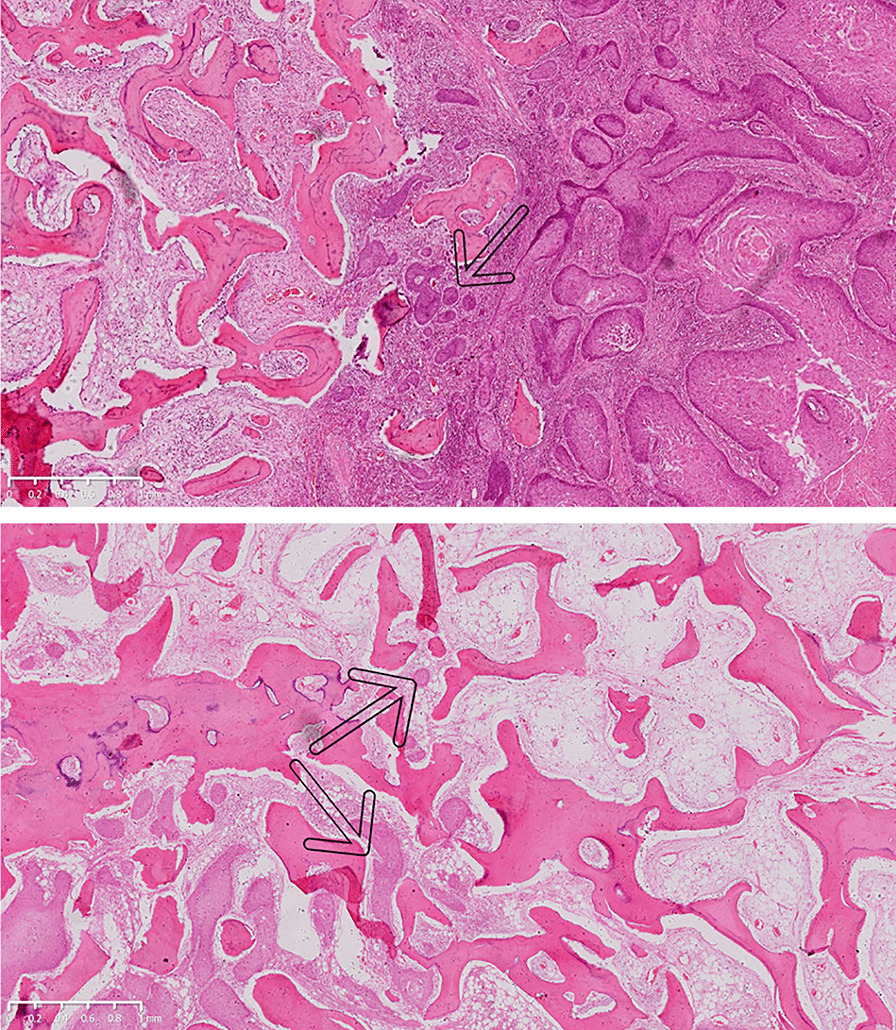
Histopathological examination of mandibular invasion by oral squamous cell carcinoma in hematoxylin–eosin-stained tissue. **a** The erosive pattern of bone invasion is characterized by fibrosis with many infiltrating lymphocytes (× 2.5 magnification). **b** The infiltrative pattern is characterized by the presence of several nests and bone islands within the tumor (× 2.5 magnification). The black arrows indicate tumors

Mandible invasion by OSCC can be divided into erosive or infiltrative patterns [6]. In the present study, the erosive pattern was characterized by a fibrotic interface invaded by numerous lymphocytes between the tumor and bone tissue, with no bone islands present within the tumor (Fig. 4a). The infiltrative pattern was characterized by several nests of tumor cells along an irregular advancing boundary, with bone islands within the tumor (Fig. 4b). Unfortunately, these two patterns could not be distinguished using CBCT.

## Discussion

Accurate evaluation of bone invasion and the exact boundary of bone invasion by OSCC is important for precise mandible resection during surgery. CBCT, which has a high spatial resolution and a low radiation dose, has been widely used in the oral and dentomaxillofacial regions. Although CBCT could be used in bone invasion diagnosis, to date, no studies have investigated the accuracy of CBCT in the evaluation of the bone invasion boundary. In our study, we compared CBCT images and histological slices to explore the possibility of using CBCT to preoperatively evaluate bone invasion, the boundary of bone invasion and nerve invasion. We hope this study will provide evidence for using CBCT to evaluate bone invasion with the above questions.

Previous studies have compared the extent of bone invasion evaluated with panoramic radiographs or spiral CT images with that determined via histopathological examination [7]. In a previous report, compared to that obtained in histological results, the bone invasion presented on panoramic radiographs was smaller (13 mm in width and 2 mm in depth); it was also smaller on spiral CT (5 mm in width and larger with 3 mm in depth). A systematic review compared several modalities in detecting mandibular invasion by OSCC, and the results showed that the sensitivity of bone invasion diagnosis for magnetic resonance imaging, CBCT, spiral CT and panoramic radiography was 94%, 91%, 83%, and 55% and the specificity was 100%, 100%, 97% and 91.7% for magnetic resonance imaging, CBCT, positron emission tomography/CT and panoramic radiography, respectively [8]. Czerwonka et al. compared the diagnostic efficiency of CBCT with that of conventional spiral CT and found that the sensitivity and specificity were 91% and 60% for CBCT and 86% and 68% for spiral CT [9].

In our study, the accuracy of CBCT in the diagnosis of bone invasion was 100%, which was higher than that in previous studies. The high accuracy may be attributed to our study using in vitro samples. However, these results still demonstrate that CBCT is a reliable tool in the diagnosis of bone invasion. For the bone invasion boundary, our study revealed an average underestimation of 2.97 mm using CBCT compared with histological slices. Considering the relatively accurate assessment of the extent of bone invasion using CBCT, precise surgical guide plates may be used in the future. To avoid recurrence, enlarged resection may be needed based on preoperative evaluation using CBCT. Moreover, in this study, we found that CBCT could not predict inferior alveolar nerve invasion with high accuracy. Nerve invasion could not be detected directly due to the poor presentation of soft tissues on CBCT. Nerve invasion was determined by discontinuity of the mandibular nerve canal, and this is an indirect sign. For some OSCCs, the infiltrated tumor cells may have reached the nerve, but the mandibular nerve canal seems intact on CBCT images because the special resolution is only 0.25 mm.

In our study, the bone specimens exhibited significant linear changes during histopathological examination. During histological processing, tissue shrinkage occurs as a consequence of fixation and the subsequent serial dehydration and rehydration procedures [10]. Buytaert and colleagues reported a bone volume shrinkage rate of 17% during tissue processing [11]. However, our study revealed more details of these changes, including shrinkage and enlargement. Previous reports have described the high significance of OSCC margin discrepancies after resection and specimen processing, as these might influence the adequacy of resection [12, 13]. Therefore, bone shrinkage should be considered in studies involving the sectioning of bone for histopathological examination. Our findings may promote improvements in the accuracy of pathology-based research.

GP points played an important role in our research. The three GP points embedded in the samples not only enabled the pathologist and radiologist to focus on the same locations within samples but were also utilized as markers to decrease the influence of shrinkage. As GP points were flexible and were inserted into the bottoms of the tissue holes, they could remain firmly in place until the specimen was sectioned. Accordingly, the GP points are superior to markers such as metallic pins, which shift easily during histopathology processing. Thus, GP points may be a very useful tool in imaging research. However, this method has shortcomings. For example, the pathological examination used 4-μm-thick sections, which were considerably thinner than the GP points. This defect could have led to errors in the merged images. Nevertheless, the differences between various planes that included GP points were very small. Although this technique is prone to error, it also yields substantial improvements.

As mentioned earlier, mandibular invasion by OSCC can be erosive or infiltrative [14–17]. The erosive pattern is characterized by a broad advancing boundary, with a well-defined interface between the tumor and the bone. Osteoclastic bone resorption and fibrosis are typically evident along the advancing boundary and support the absence of bone islands within the tumor mass. In contrast, the infiltrative pattern is characterized by nests and projections of tumor cells along an irregular advancing boundary, residual bone islands within the tumor, and Haversian system penetration. The presence of features of both patterns suggests a mixed-pattern invasion. Unfortunately, we did not observe distinguishing features related to these invasive patterns on CBCT. Therefore, the improvement of preoperative examination techniques remains a huge challenge.

The validation of medical imaging tools is an area of great clinical interest, and highly accurate coregistration between histopathological and radiological images in terms of the tumor boundaries can provide further clarity. The findings of this study suggest that researchers should consider bone shrinkage due to histopathological processing as a means of improving the accuracy of future bone studies. GP points can be utilized as markers to decrease the influence of shrinkage. Moreover, CBCT is a reliable and highly accurate method for predicting mandibular invasion but is considerably less accurate for the estimation of nerve invasion. The calculated underestimation of invasion was 2.97 mm on CBCT, which was lower than previously reported values. This suggests enormous potential for narrowing the extent of mandibulectomy for mandibular preservation.

## Conclusion

CBCT is quite reliable in determining the presence or absence of mandible invasion but not as reliable for nerve invasion. The deviation in bone invasion boundary estimation should be considered in osteotomy for OSCC.

## Data Availability

The data that support the findings of this study are available from the corresponding author, but restrictions apply to the availability of these data. Data are, however, available from the authors upon reasonable request.

## Abbreviations

CBCT: Cone-beam computed tomography
OSCC: Oral squamous cell carcinoma
GP: Gutta-percha
